# A Bayesian belief data mining approach applied to rice and shrimp aquaculture

**DOI:** 10.1371/journal.pone.0262402

**Published:** 2022-02-09

**Authors:** Marcus Randall, Andrew Lewis, Ben Stewart-Koster, Nguyen Dieu Anh, Michelle Burford, Jason Condon, Nguyen Van Qui, Le Huu Hiep, Doan Van Bay, Nguyen Van Sang, Jes Sammut

**Affiliations:** 1 Bond Business School, Bond University, Gold Coast, Queensland, Australia; 2 Institute for Integrated and Intelligent Systems, Griffith University, Nathan, Queensland, Australia; 3 Australian Rivers Institute, Griffith University, Nathan, Queensland, Australia; 4 School of Agricultural, Environmental and Veterinary Sciences, Charles Sturt University, Wagga Wagga, Australia; 5 Department of Soil Science, College of Agriculture, Can Tho University, Can Tho City, Vietnam; 6 Research Institute for Aquaculture No 2, Ho Chi Minh City, Vietnam; 7 Centre for Ecosystem Science, School of Biological, Earth & Environmental Sciences, The University of New South Wales, Sydney, Australia; Duy Tan University, VIET NAM

## Abstract

In many parts of the world, conditions for small scale agriculture are worsening, creating challenges in achieving consistent yields. The use of automated decision support tools, such as Bayesian Belief Networks (BBNs), can assist producers to respond to these factors. This paper describes a decision support system developed to assist farmers on the Mekong Delta, Vietnam, who grow both rice and shrimp crops in the same pond, based on an existing BBN. The BBN was previously developed in collaboration with local farmers and extension officers to represent their collective perceptions and understanding of their farming system and the risks to production that they face. This BBN can be used to provide insight into the probable consequences of farming decisions, given prevailing environmental conditions, however, it does not provide direct guidance on the optimal decision given those decisions. In this paper, the BBN is analysed using a novel, temporally-inspired data mining approach to systematically determine the agricultural decisions that farmers perceive as optimal at distinct periods in the growing and harvesting cycle, given the prevailing agricultural conditions. Using a novel form of data mining that combines with visual analytics, the results of this analysis allow the farmer to input the environmental conditions in a given growing period. They then receive recommendations that represent the collective view of the expert knowledge encoded in the BBN allowing them to maximise the probability of successful crops. Encoding the results of the data mining/inspection approach into the mobile Decision Support System helps farmers access explicit recommendations from the collective local farming community as to the optimal farming decisions, given the prevailing environmental conditions.

## Introduction

Farmers the world over are facing increasing challenges to production due to changing environmental conditions, many of which are outside their control. These environmental conditions include changing patterns of temperature and precipitation due to climate change as well as changes to the availability of water from upstream resource development [1]. In addition to these large-scale factors there are also declines in more local-scale conditions such as on farm soil and water quality [2]. These local-scale changes may be due to decisions made by farmers, such as inappropriate fertiliser application or mismanagement of crop rotation [3]. Equally, local-scale changes can occur due to factors outside the control of farmers, such as water quality reduction from nutrient inputs from nearby farms [4]. Collectively, these challenges to production can lead to crop failure and have a subsequent negative impact to livelihoods [5].

With comparatively limited capacity to absorb years of poor production, smallholder farms in developing regions are particularly vulnerable to a range of agricultural pressures. While crops are sold for cash, on-farm production also provides an important source of food for the household [6]. As such, it is crucial to ensure, as much as possible, that there is a low risk of crops failing [7] as the effects could be devastating. In many regions of the world, farming practice is driven by experience and information handed down from previous generations or those in the community. However, advances in decision support technologies and data analytics provide an avenue for technology-assisted decision making and practice, even in data poor regions [8]. One such approach, known as Bayesian Belief Networks (BBNs) [9], relies on sets of propagating probabilities of causal relationships through an influence diagram that reflects uncertainties in natural systems. As such, BBNs have been shown to be highly effective for decision making for agricultural, ecological and environmental problems [10].

A BBN is a directed acyclic graph comprising nodes and links that collectively describe a given system [11]. The direction in the acyclic graph indicates the direction of causality within the network with “parent nodes” connecting to “child nodes” according to causal relationships [11, 12]. In a BBN, the nodes represent processes or factors within the system and each node has a set of mutually-exclusive levels, known as states. The probability of each state occurring is dependent on the state of any parent nodes [12]. These probabilities are contained within conditional probability tables (CPTs) and can be defined by available data or different sources of expert knowledge [11]. In effect, any ultimate parent node (or “root node”) is considered input to the problem, whereas an ultimate child node (or “leaf node”) is an output. An important aspect of BBNs is D-separation, which means nodes that are separated by another node are conditionally independent [11]. Theoretically, these networks may be of unlimited size and complexity in terms of the number of nodes and links. For an in-depth general discussion of BBNs, see, for example, Pearl [9].

Due to their wide utility in modelling probabilistic systems and decision support, BBNs have been applied on a number of occasions to ecological, environmental and agricultural systems [7, 13–15]. For example, Baran, Jantunen, Chheng and Hoanh [16] developed BBN-based decision support systems for water management in situations of conflicting, conjunctive water use in agriculture in the Mekong delta. Similarly, Newton, Marshall, Schreckenberg, Golicher, te Velde, Edouard, and Arancibia [17] developed a BBN to predict the probability of growing non-timber products in forested areas successfully, to ensure the livelihoods of subsistence forest workers. While BBNs have proven useful in agricultural settings, particularly in knowledge rich but data poor contexts, they do come with their own limitations [18].

Many challenges with using BBNs have been documented elsewhere, such as the restriction to categorical variables and the difficulty integrating feedback loops. An additional complication is delivering them to potential users in an accessible form. It is not uncommon for BBNs developed for complex agricultural problems to themselves become very complex. For example, the BBN developed for rice-shrimp farming on the Mekong Delta by Stewart-Koster et al. [7] comprises 33 nodes, 46 links and 1,140 conditional probabilities. To use that BBN to guide on-farm decision making, observations of prevailing environmental conditions need to be entered into proprietary software and every permutation of potential decisions then need to be explored individually. Alternatively, Bayesian Decision Networks can be developed. However, these models rely on data quantifying the cost of interventions in the network and the utility of outcomes (e.g., Phan et al [19] and Stewart-Koster et al. [7]). In addition to the reliance on proprietary software, without complex cost and income data a standard BBN such as that in Stewart-Koster et al. [7] does not provide explicit recommendations of the optimal decision given the prevailing environmental conditions. As such, a specialised approach is needed to interrogate the model, particularly in the context where proprietary software may not be available to end-users, such as smallholder farmers on the Mekong Delta, to enhance the utility of a given BBN in the hands of an end-user. Novel data mining approaches that can be integrated into generic, smartphone-based software may be useful to interrogate a BBN and provide direct recommendations of optimal decisions.

In this paper, a novel temporally-inspired data mining approach for BBNs is presented. Working with an existing BBN that describes a combined rice-shrimp farming system commonly used on the Mekong Delta [7], an approach to extract all possible scenarios that may occur and identify the optimal decision from the myriad options available to them, is developed. This approach combines a novel temporally-oriented data mining system with visual analysis to provide explicit automated decision support in a framework that can be embedded in a freely available format for smartphones.

To demonstrate the concepts of temporally-inspired data mining and automated visualisation, an existing BBN that stores the knowledge of farmers for optimising crop and shrimp yields in the same pond in the Southern Mekong Delta, Vietnam [2, 7], is used. Preliminary exploration of the use of a BBN for rice/shrimp farmers [20] found that it was a viable method to help make sensible decisions, though more work was needed to realise its potential. Researchers initially worked actively with farmers and extension officers to elicit and establish relationships between different factors that affect the success of the rice and shrimp pond systems. The conditions include, but are not limited to, factors such as environmental conditions (e.g., rainfall), stocking densities and the application of various types of fertilisers. This has been encoded as a BBN, and from it, it is possible to derive sets of conditions with appropriate decision values that reduce the probability of crop failure, for both rice and shrimp. The systematic interrogation of the BBN at various stages of the crop cycle is important to allow farmers to make the best possible decisions in a timely manner. A simple data mining approach with visual analysis of the extracted data is shown. Observations from the visual analysis allow for the development of automated post-processing of data at each stage. Using this multi-stage classification approach of decisions and actions that farmers need to take, a practical decision support system is developed.

## A data mining approach applied to the rice/shrimp BBN

In this section, a novel temporally-inspired data mining approach for BBNs is presented. The two essential components for helping farmers to make informed decisions to ensure good harvests are the BBN and the novel temporal data mining approach developed here. Both are described below.

### The subject BBN

**Fig 1:**
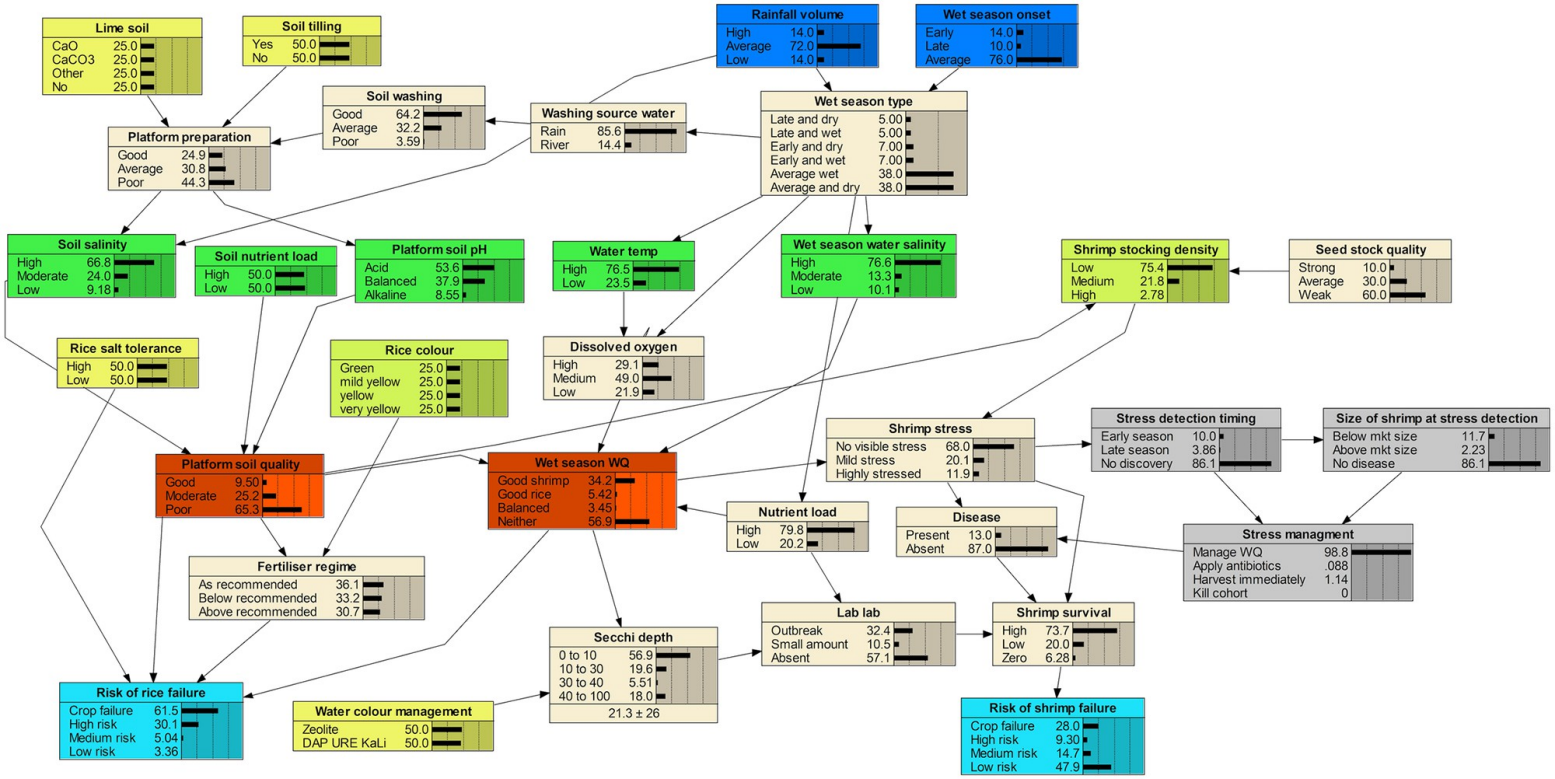
The rice/shrimp cultivation BBN as used by Stewart-Koster at al. [7], and as a basis for this paper. Reproduced from Stewart-Koster [7], p. 235.

The data mining system has been designed to integrate a standard BBN, similar to the one developed, and previously described by Stewart-Koster et al. [7]. This network described a combined aquaculture-agriculture system in which rice and shrimp crops are simultaneously grown in the Mekong Delta in Vietnam. Rice-shrimp systems have become relatively widespread in parts of the developing world, becoming an important farming system in Vietnam [2], and consist of a pond with a raised soil platform surrounded by a narrow, but deep water-filled ditch. The rice is planted on the platform while the shrimp mainly inhabit the ditch, but feed on the smaller invertebrates that consume the algae that grows on the sediment, the rice plants and rice stubble [21]. Both crops require very different conditions to return high yields, with salinity levels being the most obvious issue when growing a brackish-water animal on the same farm as a freshwater plant. While the two crops are grown in rotation, it is increasingly common that farmers will grow them concurrently, for at least some of the season, and creating tolerable conditions for both is the major challenge faced by the farmers [2]. The BBN in Stewart-Koster et al. [7] was designed to capture the perceptions of farmers and extension officers of key risk factors to production and their understanding of how the system works. For reference, this BBN is reproduced in Fig 1.

The process to develop the BBN was based on the Iterative Bayesian Network Cycle [13] where experts are consulted in multiple iterative workshops involving elicitation and validation. These workshops occurred over three research trips between October 2013 to November 2015 with each set of workshops involving distinct phases of BBN development as follows:

1. Identifying key processes and causal relationships,
2. Defining the node states and the conditional probabilities withing the BBN and
3. Verification of the final network.

These workshops included farmers and local policy extension officers from three provinces of the southern Mekong Delta (Ca Mau, Bac Lieu and Kien Giang) and each phase involved a series of elicitation and validation steps to ensure the final network represented the collective knowledge and experience of the participating farmers across the region. A full description of the iterative process used to develop the BBN, including the validation/verification of the network itself, is provided in the Materials and Methods section of Stewart-Koster et al. [7]. In the current study, no new data is added to this BBN, but rather extracted from the complete data set of probable outcomes embodied within it for systematic analysis.

It is important to note that the BBN was not intended to predict crop yield. Rather the network was designed to quantify the perceptions of important risk factors that contribute to the probability of crop failure, as discussed in Stewart-Koster et al. [7]. As such, many of the nodes and the thresholds of the nodes do not have an objectively measurable basis. For example, the smallholder farmers participating in the information gathering workshops do not have access to scientific equipment to measure such things as salinity. In that case, farmers taste the water to determine if it is “good for rice” or “good for shrimp”. Consequently, verification involved confirming that the probable outcomes in the BBN, given the scenarios in the parent nodes, reflected the farmers’ understanding and experience rather than the network predicting certain outcomes that could be measured by scientific equipment. While the BBN itself provides a semi-quantitative description of the collective understanding of the system, it is not currently accessible to the farmers of the Mekong delta, as potential users need to have access to proprietary software and since it does not include cost and utility processes, it lacks the capacity to identify optimal decisions.

### The temporal data mining approach

In order to secure useful outcomes for farmers, based on the network in Fig 1, a data mining approach is employed to extract recommendations encoded in the BBN, without the need for proprietary software that is essentially unavailable to the farmers. This is based on understanding the network and finding patterns that occur in it [22]. It is possible to identify optimal decisions in a Bayesian Decision Network [15], however, in the absence of monetary cost and utility data to complement the BBN, an alternative approach is needed. The two output nodes of the network are “Risk of rice failure” and “Risk of shrimp failure”. By testing inputs to other nodes, particularly those that define the farming environment or represent actions that farmers might take, it was possible to discover the sets of conditions and decisions that minimise the probability of rice and shrimp failure. This was done by selecting one of the possible input conditions for a node and setting the probability of that condition to 100%. Hence, the other possible input probabilities for that conditions become 0%. Thus there is a finite and limited set of combinations to explore, given a prevailing set of environmental conditions. Many nodes are considered to be intermediate (not requiring a human input decision, or calculating a final output), therefore cutting down the size of this exploration considerably.

Inspection of Fig 1 shows that the network spans the planting/harvesting cycle for both shrimp and rice. At different stages, certain actions are, or are not, possible or practical. For example, it is not possible to till the soil after planting. Therefore, some sensible temporal decomposition of the network is possible. This gives three phases, namely, *pre-planting*, *planting* and *post-planting*. These are referred to as *stages*. Within each stage, environmental conditions, or actions already taken, define one of a number of *scenarios*. These conditions or actions are set to a probability of 100% in the relevant nodes. This has the effect of nullifying any input from any parent nodes. Each of the stages and their defining conditions are described in depth below:

1. *Pre-planting*—The CPTs “Rainfall volume” and “Wet season onset” define the climatic conditions for the season. These factors along with “Soil nutrient load” help to determine which actions must be taken by the farmer before the rice crop is sown and the shrimp put in the pond. In Fig 1 the nodes that encapsulate these CPTs (the dark blue nodes for climatic conditions, and the green node for Soil Nutrient Load have their states fixed according to the chosen scenario. For example, the probability that the Rainfall Volume was “High”, the Wet Season Onset was “Early” and the Soil Nutrient Load was “High” may be set to 100%, selecting the corresponding scenario.) Farmers can then choose to apply liming agents to the soil, and whether or not to till the soil. Other factors that may affect the final outcomes will be what shrimp stocking density they choose, whether they decide to plant salt-tolerant rice, and what water colour management option they use.
2. *Planting*—At this point, it is still possible for farmers to make a series of estimations. These include measures of “Soil salinity”, “Platform soil pH”, “Water temperature”, “Wet season water salinity” and “Soil nutrient load”. These are the green nodes in Fig 1. Specifying the state of these nodes, by fixing the state probability to 100% for a chosen state, removes the effect of their parent nodes, such as wet season timing, because of D-separation. Having made these estimations, farmers’ actions are now limited to whether to choose salt-tolerant rice, what stocking level of shrimp to choose, and the water colour management option.
3. *Post-planting*—After the rice has been planted and the shrimp stocked in the pond, a new set of nodes, lower in the BBN, has been defined, further reducing the number of options available to the farmers as the season progresses. The scenario is defined by “Platform soil quality” and “Wet season W[ater]Q[uality]”, the orange nodes in Fig 1, and what choices were made for rice salt-tolerance or shrimp stocking density. However, it is desirable to keep the state probabilities chosen for the higher level nodes from the planting season scenario because these are based on physical measurements, rather than subjective assessment: for example, whether “wet season water quality” was good for shrimp. To these are added what actions were taken at planting, i.e., choice of rice variety and shrimp stocking density. The corresponding nodes are set to the state chosen at planting. Farmers’ actions are now limited to assessing rice colour (as part of the scenario), which will determine the level of fertiliser applied, and choosing the water colour management treatment for shrimp production.

As is evident from the above, this form of decomposition allows farmers to make a number of structured choices, gradually decreasing in flexibility as the season progresses. However, this is designed to help farmers make good decisions at each stage of the growing cycle. In general, for this style of technique to be able to be applied, the context of the BBN must have definite temporal divisions (scenarios). By their very nature, agricultural applications, which comprise event based decisions over time, are very amenable to this approach.

## Computational experiments and results

To investigate the three different stages during the growing season, three computational experiments were conducted. For each stage, the BBN was run by entering discrete conditions for the nodes defining each scenario. Then permutations of the possible actions and choices were entered for each scenario. In terms of the implementation to achieve this, a wrapper program in C was developed. It repeatedly ran Netica (Version 4.16 [23]) through an Application Program Interface (Netica_API_504) to achieve this systematic exploration of all enumerations for the three scenarios. The data gathered through this program for each of the experiments represents a complete enumeration of a decision tree [24]. A separate dataset was delivered for each experiment, corresponding to one of the three growing season stages. The corresponding belief of the probability of failure of the rice and the shrimp crops was recorded for each combination of choices in each scenario.

Fig 1 includes a number of actions responding to shrimp stress and disease. While these actions significantly impact shrimp survival rates, they are critically dependent on a number of unknown factors, such as the timing of detection of shrimp stress. For this reason, these factors were not included in this particular study.

**Fig 2:**
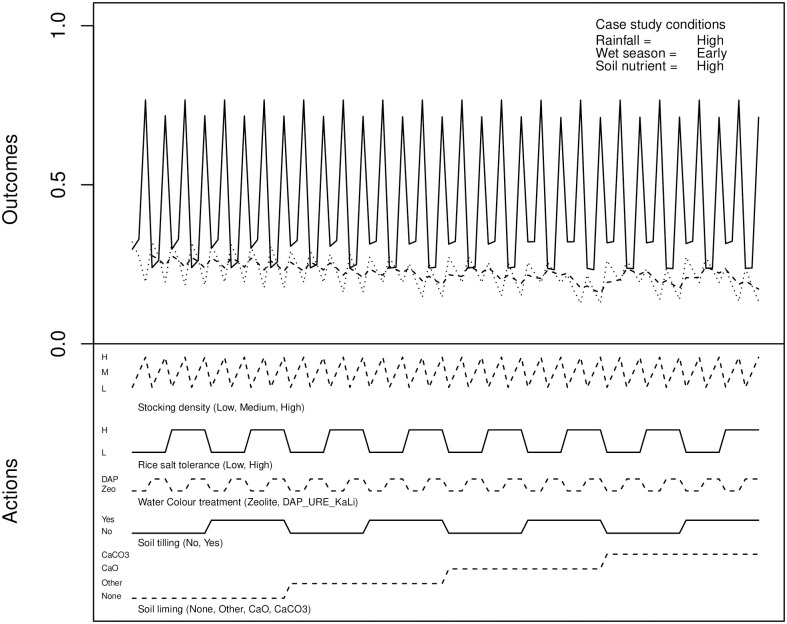
An example of the raw output of the pre-planting experiment.

In order to determine optimal actions for crop management, and relationships between actions and outcomes, a visual analytics approach [25, 26] was used. The data from each individual scenario in each growing stage were visualised using the R statistical analysis language [27]. An example of the raw data presentation is shown in Fig 2.

This figure illustrates data obtained for one scenario in the pre-planting stage. This stage is governed by evaluation of climatic conditions (the arrival time of the wet season, and intensity of rainfall) and the condition of the soil (whether soil nutrient load was high or low.) The corresponding nodes in the BBN had probabilities set to 100% for one of their states, defining one of 18 possible scenarios. The states chosen are shown in the upper-right of the figure.

The varied actions possible in this particular scenario are shown in the lower traces, the probability of shrimp crop failure (solid line) and rice crop failure (dotted line) in the upper traces. For example, the probabilities of crop failures in the case of using high shrimp stocking density, low rice salt tolerance, tilling the soil, applying no soil liming and using Zeolite to control water colour can be found finding the points on the upper traces that correspond to these settings in the lower traces.

**Fig 3:**
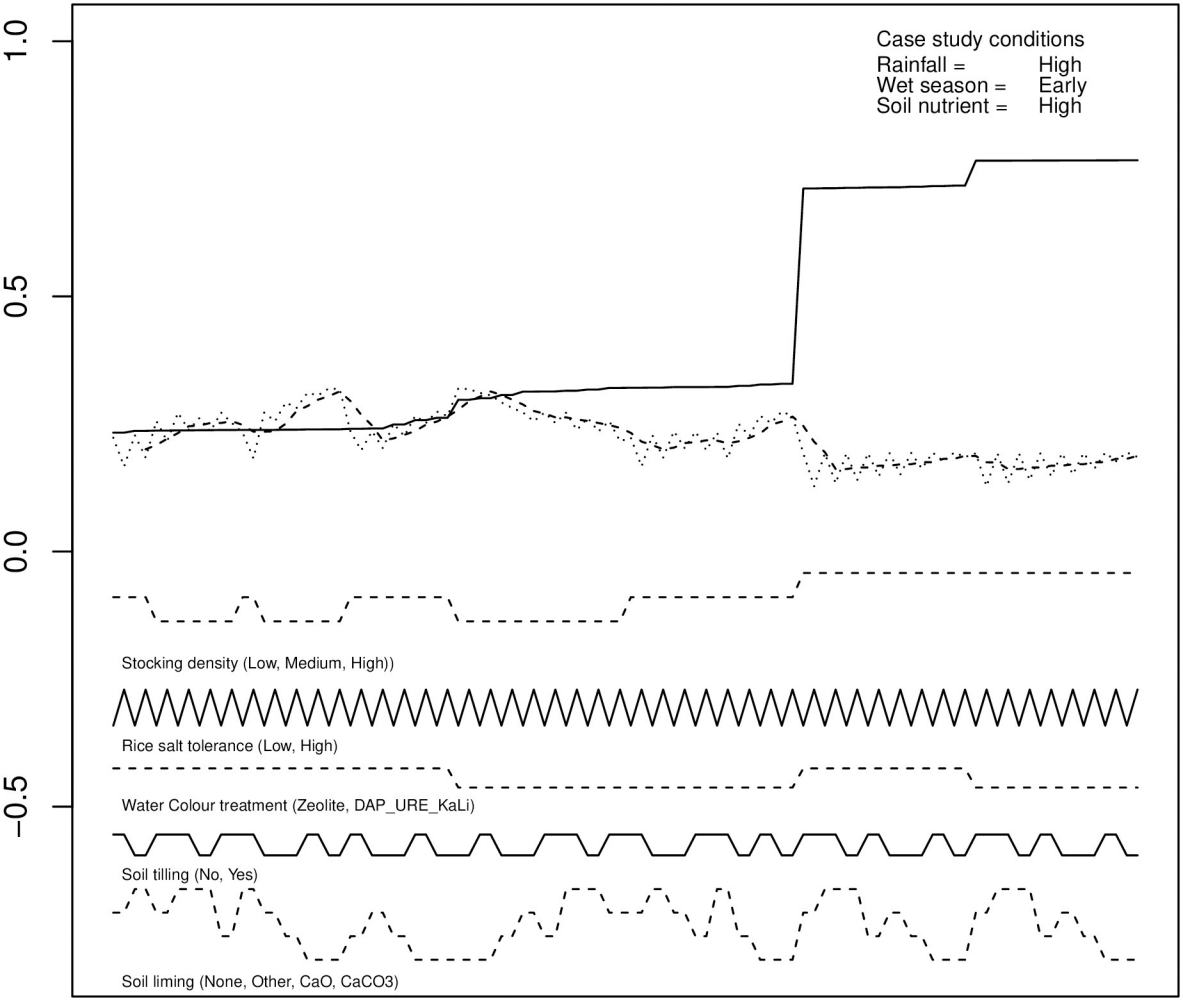
An example of the pre-planting experimental output, sorted by probability of shrimp crop failure.

Both traces for crop failure probabilities are quite “noisy”. It should be noted these are traces of discrete data, not continuous functions. Only stationary or flexion points correspond to meaningful data. While the probability of rice crop failure shows some indication of trends, corresponding to some extent with soil treatments, as shown by the superimposed simple moving average (dashed line), the same cannot be said of the shrimp crop failure. The plot does appear to indicate that the values for probability of shrimp crop failure tend to cluster around a small number of discrete values. For this reason, the dataset was reordered based on these values. The resulting plot can be seen in Fig 3.

From Fig 3 the root causes of shrimp crop failure, largely that of overstocking, can now be identified. It is also evident that a compromise of near-minimal probability of failure of both crops can be found by searching for the first low point of probability of rice crop failure in the re-ordered data (which will also correspond with a low probability of shrimp crop failure). This was a pattern that appeared across all of the scenario data subsets. This simple process of inspection can be automated through the same R script used to sort and present the data. Essentially the R script mimics a visual analysis of the data.

Three corresponding scripts were developed to extract the data for a given scenario, reorder the data, and determine the set of conditions for the case satisfying mutually minimal probability of failure of the rice and shrimp crops. The scripts output this information in text and graphical form.

**Fig 4:**
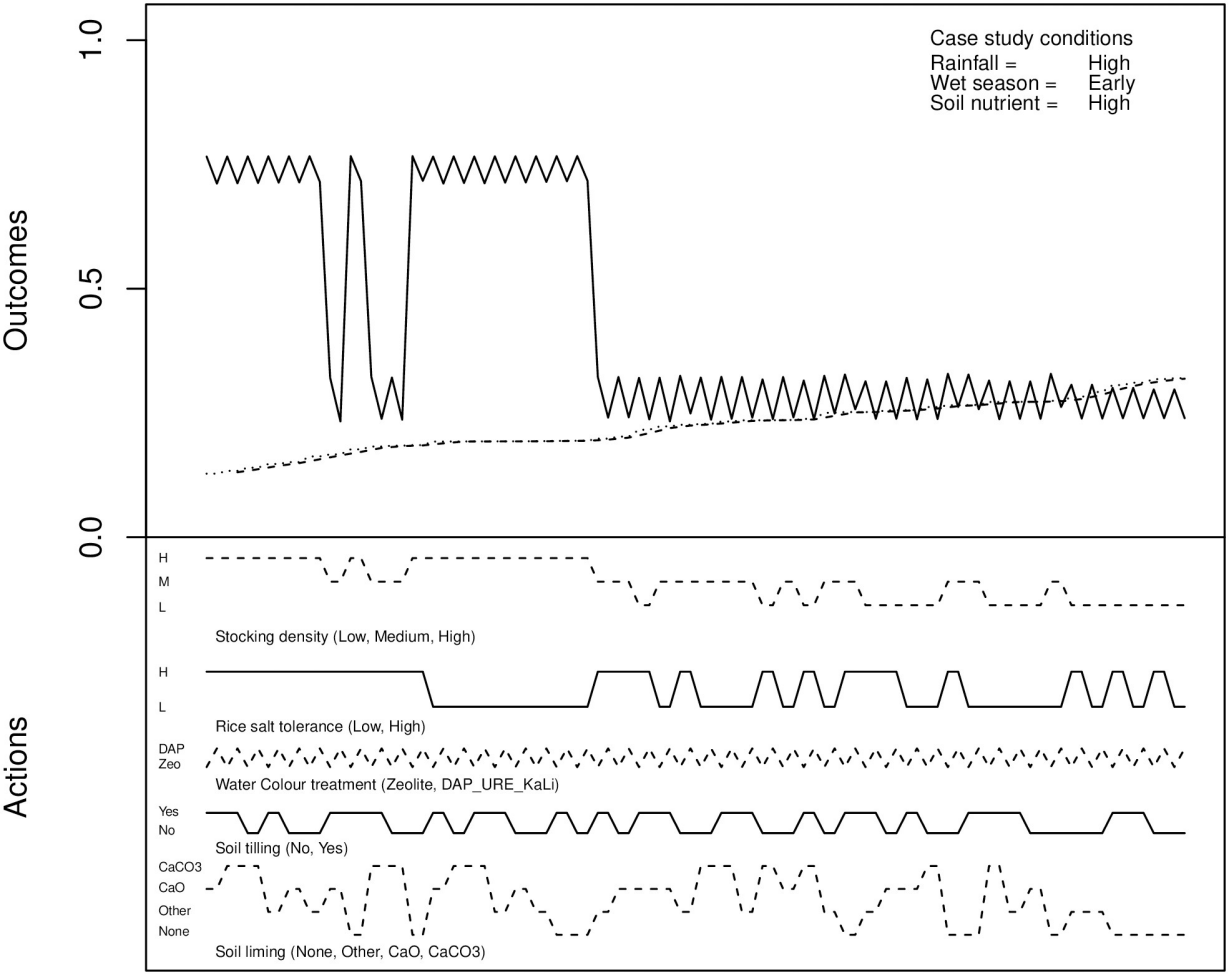
An example of the pre-planting experimental output, sorted by probability of rice crop failure.

It might be said that this approach prioritises avoiding shrimp crop failure—from Fig 3 it can clearly be seen that slightly lower probability of failure of the rice crop could be achieved by other actions (toward the right-hand end of the plots.) Though this does come at the expense of significantly increased probability of failure of the shrimp crop, there may be cases where such an outcome is justified. If this was desirable, the data could be initially reordered based on rice crop outcomes, as shown in Fig 4.

As was noted earlier, the probability of better outcomes for the rice crop come at the expense of the shrimp crop. Early permutations in the reordered dataset are quite poor for shrimp. It takes more careful searching of the data to identify a combination of actions that present a satisfactorily low probability of shrimp crop failure, but this could be found automatically by placing a threshold on acceptable probability of failure.

**Fig 5:**
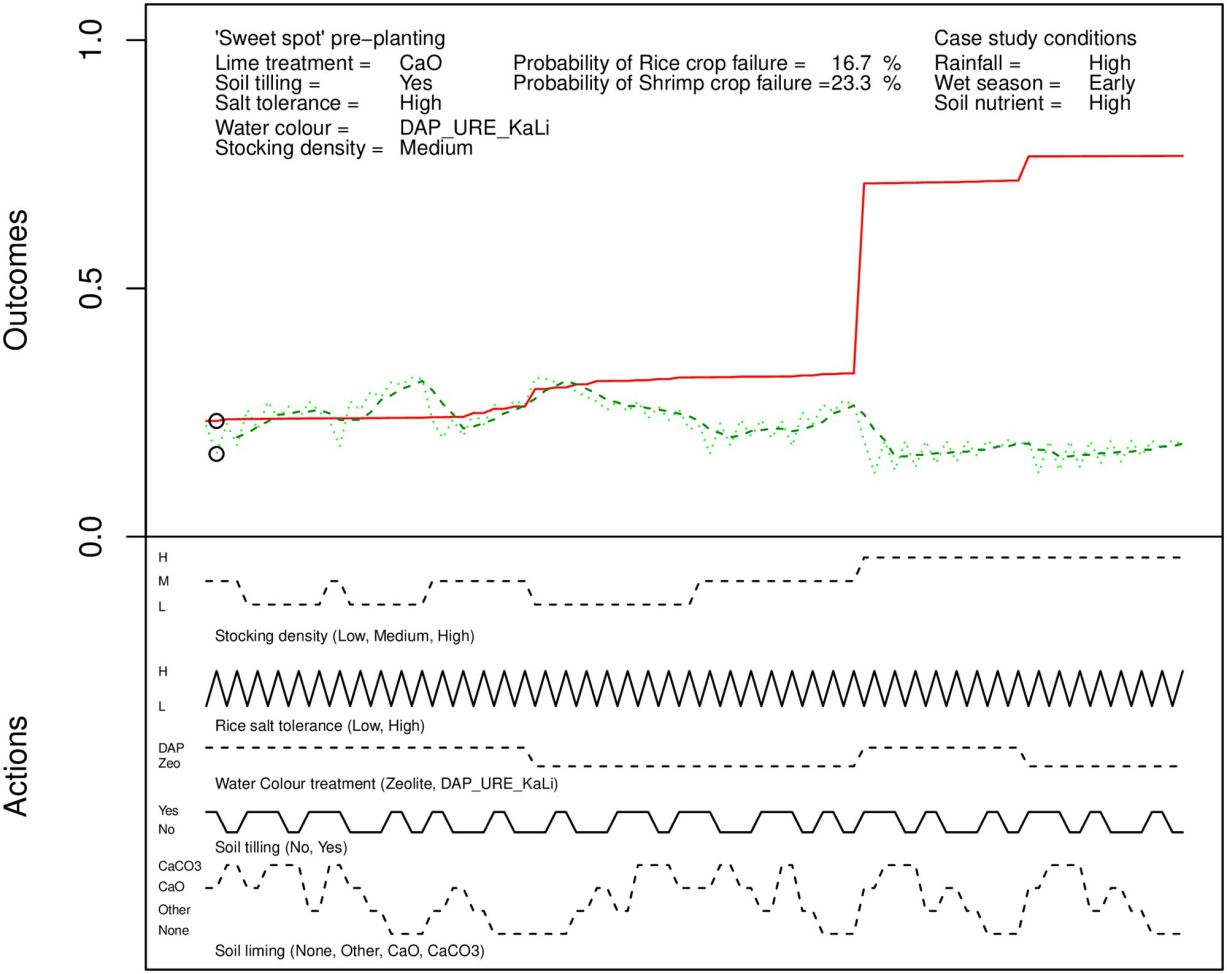
An example of the post-processed pre-planting experimental output.

An example of the final output from the pre-planting analysis is shown in Fig 5. The figure shows the optimal actions for a scenario of early onset of the wet season with heavy rainfall and high soil nutrient load.

The “sweet spot” for the best outcomes has been determined algorithmically and circled on the graphs of probability of rice crop failure (green line overlaid with simple moving average smoothing) and shrimp crop failure (red line). The scenario conditions are provided for reference at the top right. The conditions to achieve the minimal probability of failure in each crop are given at the top left. It is clear that the farmers perceived it is optimal to till the soil and apply CaO (quicklime). The actions that were perceived to achieve the corresponding probabilities of crop failure are graphed directly below. These allow exploration of “what if” variations of decisions to illustrate the expected change to probability of crop failure if a farmer made different decisions from those identified as optimal. Consider the case when the shrimp crop density is increased to “high” (labelled dotted line below the main graph, toward the right). It can clearly be seen that there is a corresponding large increase in the probability of shrimp crop failure.

**Fig 6:**
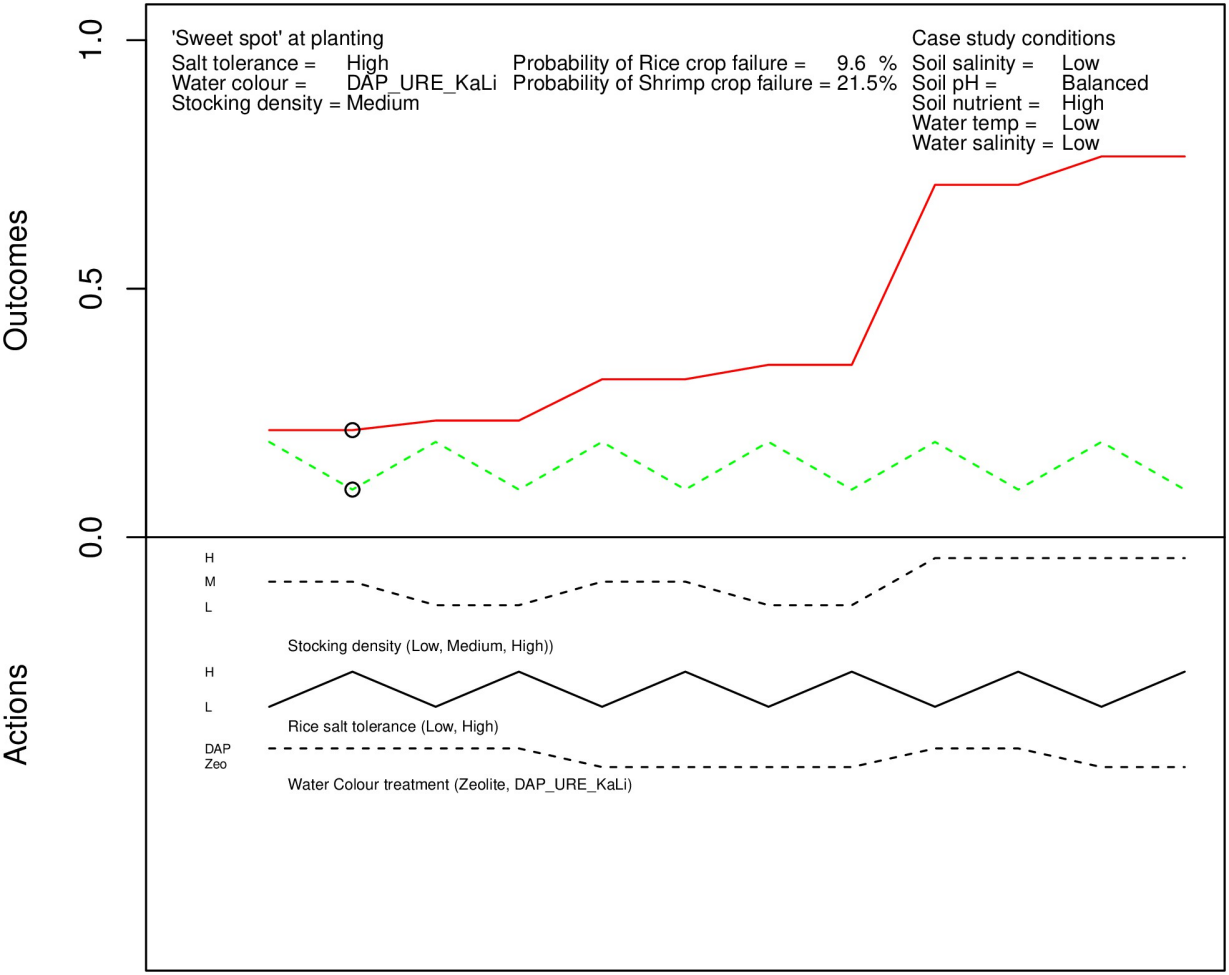
An example of the planting experimental output.

An example of output for the second stage, the planting season, is shown in Fig 6. The scenario shown is for low soil and water salinity, balanced soil pH, high soil nutrient load and low water temperature. As can be seen, there are fewer actions available to farmers. The graphs have fewer distinct cases within the scenario, though there are more discrete scenarios. As before, the link between stocking density and shrimp crop failure is evident.

**Fig 7:**
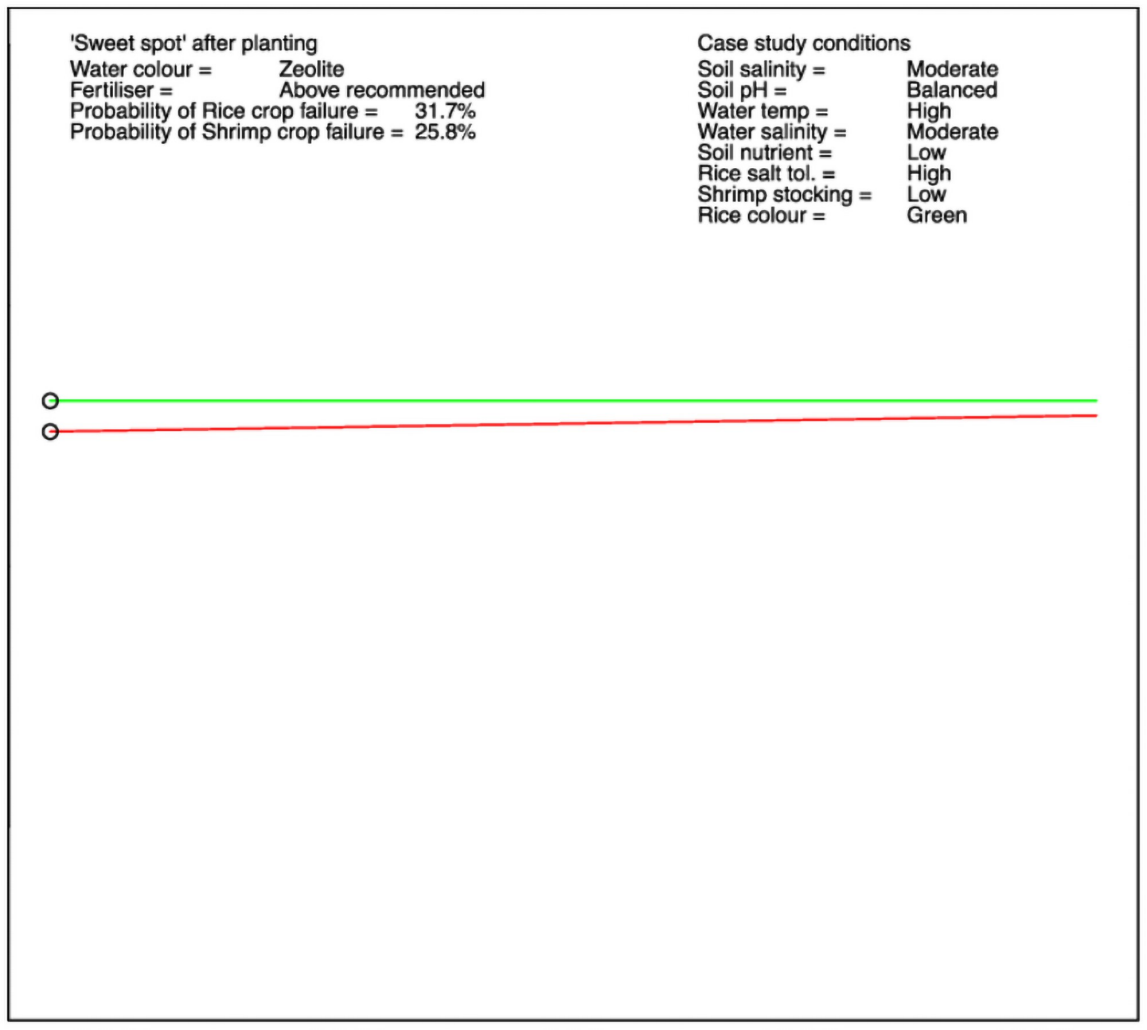
An example of the post-planting experimental output.

For the final stage, post-planting, an example is shown in Fig 7. The scenario is now defined by eight conditions, and there is only one choice available to farmers: the “water colour management” option. The optimal actions also mention that fertiliser applied should be above the recommended level. However, this is not a “free” choice –- it is determined by the farmer’s assessment of the rice colour (see Fig 1), and the factors contributing to the “Platform soil quality”, governed by the choice of scenario. It must be extracted from the BBN data by searching for the fertiliser level with the highest conditional probability corresponding to the optimal actions within the scenario. This highlights a consideration that should ideally be kept in mind when designing BBNs –- nodes defining actions to be chosen should not have parent nodes that constrict their conditional probabilities. While there are only two states within each scenario depending on the “water colour management” choice, there are over 2500 distinct scenarios of detailed specification.

In the systematic exploration of the causes and effects described by a BBN shown in this paper, three key ideas have been employed:

1. *Fixing the (discrete) values for a set of root nodes of the BBN to describe a “scenario”*. By doing this, discovering optimal actions to achieve desired outcomes, in the context of particular scenarios, becomes more tractable as only a subset of all possible permutations need be considered.
2. *Truncating the decision tree to reduce the quantity of data to be explored*. The BBN, as a directed acyclic graph, can also be interpreted as a representation of a decision tree. By selecting a “tree-wide” subset of nodes to describe a scenario, it is possible to remove the effects of antecedent (parent) nodes. This has the effect of reducing the number of permutations within a scenario (while inevitably leading to an increased number of those scenarios) further easing the search for optimal decisions within a scenario. For the agricultural problem considered in this work, considering separate stages in the growing season made the selection of the nodes for truncating the decision tree quite obvious.
3. *The use of a visual analytics approach to interpret the data*. As has been shown, even with the preceding simplifications the data representing permutations and corresponding outcomes can be “noisy” and difficult to interpret. By plotting and viewing the data, a simple but effective data visualisation, insight can been gained into relationships between cause and effect. This can further lead to the ability to codify the insight in automated scripts for data post-processing. It should be noted that introducing the “human-in-the-loop” [28] using visual analytics has enabled experiential learning, and allowed the development of simple but effective methods of data processing and knowledge extraction.

## Conclusions

The work in this paper described a data mining and visual analytics approach to the analysis of BBNs. Knowledge was extracted computationally by exhaustive enumeration, visually analysed, and automated data post-processing techniques subsequently developed.

The advantage of BBNs as an approach to decision support for farmers is that it is a form of participatory modelling that engages them as experts in model development. This ensures the model underpinning the decision support system reflects the current understanding that system which helps to engender belief in the system. Moving from a standard BBN that describes a system and answers questions such as “What happens if a particular action/decision is taken?”, to a probabilistic DSS that answers questions such as “What is the optimal action/decision?” requires additional data or steps. Bayesian decision networks provide an avenue to achieve this. However, when lacking cost and income data the approach used in this work provides a way to interrogate a BBN and obtain recommendations for the optimal action/decision given the prevailing conditions.

Despite the advances of this systematic data mining approach, there are nonetheless key limitations. As with any modelling exercise, the utility of the model is dependent on its quality. The accuracy of the BBN that was previously developed therefore must be relied upon. If there were errors of communication between the model developers and the farmers that led to errors in the BBN structure, the approach will only amplify them and not identify them as a problem. A potential avenue to solve this begins at the development of the BBN. This could come from using a portfolio of more sources of data coupled with consultation with a wider range of experts including farmers and scientists to refine the underlying BBN. A key objective for this work was to deliver effective decision support to smallholder farmers in the Mekong delta. To achieve this, and embed knowledge from the BBN into a usable platform for farmers, the development of a smartphone app has begun [29]. It is founded on the principles of cultural sensitivity and ease of use for the farmers in the region. These designs have already been tested, and a prototype smartphone app is ready for field deployment and evaluation. Combining our temporal data mining approach with the BBN data in contemporary smartphone technology could help farmers make decisions in the face of changing environmental conditions in the region.
