# Correction: A Bayesian belief data mining approach applied to rice and shrimp aquaculture

**DOI:** 10.1371/journal.pone.0307315

**Published:** 2024-07-11

**Authors:** Marcus Randall, Andrew Lewis, Ben Stewart-Koster, Nguyen Dieu Anh, Michelle A. Burford, Jason Condon, Nguyen Van Qui, Le Huu Hiep, Doan Van Bay, Nguyen Van Sang, Jesmond Sammut

The 5^th^ and 11^th^ authors’ names are written incorrectly. The correct names are, respectively: Michelle A. Burford and Jesmond Sammut.
